# A 5-Year Review of Invasive Fungal Infection at an Academic Medical Center

**DOI:** 10.3389/fcimb.2020.553648

**Published:** 2020-10-22

**Authors:** Yaling Li, Yali Gao, Xueli Niu, Yutong Wu, Yimei Du, Ying Yang, Ruiqun Qi, Hongduo Chen, Xinghua Gao, Bing Song, Xiuhao Guan

**Affiliations:** ^1^Department of Dermatology, The First Hospital of China Medical University, Shenyang, China; ^2^National Health Commission Key Laboratory of Immunodermatology, China Medical University, Shenyang, China; ^3^Department of Biotechnology, Beijing Institute of Radiation Medicine, Beijing, China; ^4^School of Dentistry, Cardiff University, Cardiff, United Kingdom

**Keywords:** invasive fungal infection, bacteremia, epidemic, single center, China

## Abstract

**Background:** Invasive fungal infection (IFI) is one of the most common nosocomial infections. However, data on the epidemiology of IFI and susceptibility to antifungal agents in China are quite limited, and in particular, no current data exist on the microbiological, and clinical characteristics of IFI patients in Northeast China.

**Objectives:** The purpose of this study was to provide a retrospective review of the clinical characteristics, laboratory test results, and risk factor predictions of inpatients diagnosed with IFI. Multivariate regression analysis was used to assess prognostic factors associated with the mortality of these patients.

**Methods:** We retrospectively analyzed the results from 509 patients with IFI extracted from the First Hospital of China Medical University from January 2013 to January 2018.

**Results:** Neutrophil numbers, total bilirubin, length of stay in the ICU, renal failure, use of immunosuppressants within the past 30 days, stomach tube placement and septic shock were risk factors for death from IFI. Recent surgery (within 2 weeks) and drainage tube placement did not increase mortality in these IFI patients. Increased serum levels of PCT (AUC 0.601, 95% CI 0.536–0.665, *P* = 0.003) and CRP (AUC 0.578, 95% CI 0.512–0.644, *P* = 0.020) provided effective predictors of 30-day mortality rates.

**Conclusions:** We report for the first time epidemiological data on invasive fungal infections in Northeast China over the past 5 years. Despite the limited available clinical data, these findings will greatly aid clinical health care workers with regard to the identification, prevention, and treatment of IFI in hospitalized patients.

## Introduction

Invasive fungal infection (IFI) is one of the most common nosocomial bloodstream infections (Bassetti et al., 2016; Bosch et al., 2016) and may result in invasive diseases in patients with various underlying diseases or other host factors. Invasive diseases most frequently occur in bloodstream infections (mycosis) or via secondary transmission to areas, such as the eyes, liver, spleen, bone, heart valves, and central nervous system, or deep candidiasis (Pfaller and Diekema, 2007; Arendrup, 2010). In recent years, the incidence of IFI has been increasing, especially within hospitalized patients (Fishman, 2007). The pathogens responsible for IFI are mostly opportunistic pathogens, including Candida, Aspergillus, and Cryptococcus (Falagas et al., 2010). Among these, *Candida albicans* is the most common (Fu et al., 2017). Increased incidence of IFI in hospitals is associated with increased susceptibility to IFI in immunocompromised patients, especially in patients with AIDS, hematological diseases, malignant tumors, organ transplantation or burns (Villarroel et al., 2010; Singh et al., 2016). Invasive fungi can affect various tissues and systems throughout the body, among which respiratory infections are the most common (Singh and Husain, 2003). IFI in hospitals is often a coinfection in some critically ill patients, and the basis of diagnosis must be fungal culture and pathological examination, which take a long time and are not conducive to early diagnosis and treatment (Clancy and Nguyen, 2013; Kim et al., 2013). As a result, there exists a high fatality rate in these IFI patients, which has attracted increasing attention within the medical community. Due to the limited number of studies, many aspects of IFI remain poorly understood, including clinical characteristics and prognostic factors. Most research on IFI has been conducted in the United States and Europe, while no reports on this condition exist from Northeast China. In particular, microbiological and clinical data among Chinese IFI patients are extremely scarce. There are no epidemiological data on IFI associated with bacterial infections in hospitals worldwide. In this study, we performed a retrospective review as a means to partially elucidate the microbiological and clinical characteristics of IFI patients in Northeast China, as well as to aid in the prognosis and identify related risk factors associated with this condition. Specifically, the goals of this study were to (1) record the fungal species distribution and drug resistance of blood- and sterile body fluid-isolated strains as an approach to describe the clinical features of IFI, (2) describe the clinical features of IFI in patients with bacterial infections, and (3) determine the risk factors associated with mortality and prognostic factors.

## Materials and Methods

### Patient Selection

Data for this study were obtained from the electronic medical record system, as extracted from the First Hospital of China Medical University over the period from January 2013 to January 2018. Clinical data from hospitalized patients with a diagnosis of IFI based on the presence of an effective fungal specimen in culture were used in this study. Information collected and recorded from these patients included age, sex, length of hospital stay, antibiotic application, chemotherapy, surgery, basic diseases, intensive care unit (ICU) admission, mortality and any other relevant clinical information. The patients at this hospital represent those from the entire northeastern section of China, which comprises a location where epidemic levels of IFI are believed to exist.

### Criteria for Study Inclusion

We collected all Candida, Cryptococcus, and other yeast isolates recovered from blood; other sterile body fluids, including ascitic fluid, and peritoneal dialysate fluid, pus, and tissue from patients with invasive yeast infections for inclusion in this study (2008 version of EORTC/MSG criteria). The data collected included the subject's characteristics at baseline, haematologic diagnosis and chemotherapy, risk factors for IFI, clinical features of IFI, fungal test results, antifungal prophylaxis and treatment, and survival status at discharge. The management of the patients receiving antifungal prophylaxis and/or therapy was recorded, including the date and nature of the change in treatment and survival status at discharge. Each inpatient hospitalization represented one case, and if a patient was re-hospitalized and received another round of treatment, he/she was also considered a new and separate case.

### Microbiological Test

Aseptic humoral samples (8–10 ml) were collected and cultured for 5 days. Positive samples were transferred to blood AGAR plates and then bacterial isolates and fungal isolates passed at 35°C for 48–72 h. Gram staining and microscopic examination were performed simultaneously. Strain identification (bacterial isolates and fungal isolates) was performed on a VITEK 2 Compact (Bio-Merieux SA, Marcy l 'etoile, France), and susceptibility tests were performed with use of ATB FUNGUS 3 (Bio-Merieux SA, Marcy l 'etoile, France).

The drug susceptibility tests were performed with use of ATB FUNGUS 3 (Bio-Merieux SA, Marcy l 'etoile, France). Drug susceptibility tests consisted of application of ATB Fungus 3 yeast-like fungi into a drug susceptibility test box in strict accordance with the “national clinical test operating procedures” and reagent instructions for the procedure. The minimum inhibitory concentration (MIC) value was determined according to the CLSI m27-a3 and m27-s4 antifungal susceptibility test standards. The quality control strains were Candida ATCC6258 and *Candida albicans* ATCC90028.

### Definition and Abbreviations

IFI, invasive fungal infection; ICU, intensive care unit; SDD, susceptible-dose dependent; PCT, procalcitonin; CRP, C-reactive protein; BDG, 1–3-β-D-glucan.

Prolonged hospitalization was defined as a hospital stay longer than 10 days.

### Statistical Analysis

The SPSS 20.0 software program was used for statistical analysis. Non-normally distributed quantitative data were presented as median and quartile ranges [M(P25, P75)] and analyzed with the Wilcoxon rank sum test for intergroup comparisons. Qualitative data were described using relative numbers, and the χ^2^ test was used for comparisons between these groups. Logistic regression analysis was used for multivariate analysis. *p* < 0.05 was required for differences to be considered statistically significant.

Note: ^a^ is described by median and quartile, and the statistic was the *Z-*value; other items were described as numbers (n–%), and the statistic was the χ^2^ value. ^b^ There was a negative correlation between the value and significance.

## Results

### General Information

**Figure 1:**
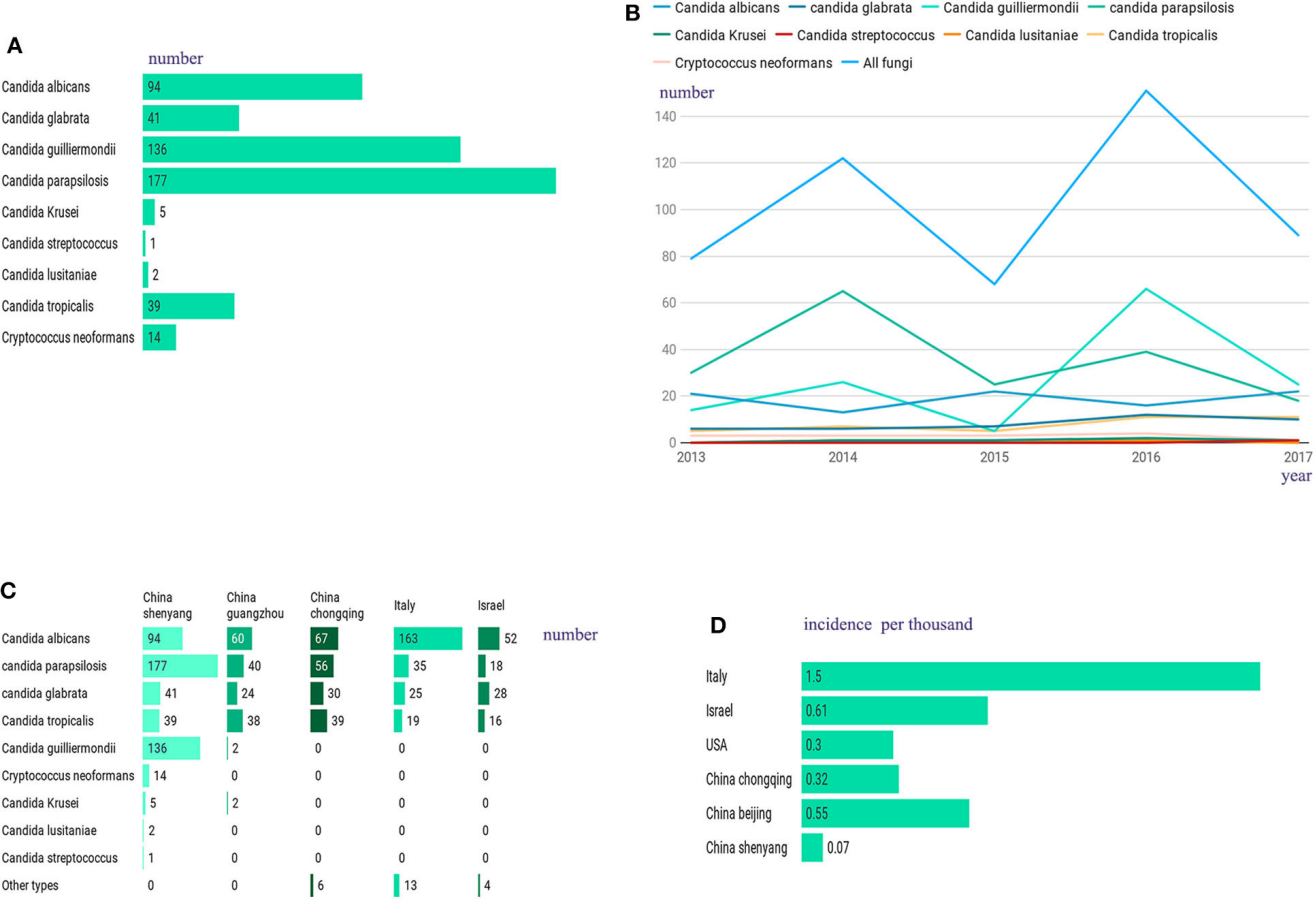
**(A)** Among the 509 hospitalized patients, the most frequent infection agent was *Candida parapsilosis* (177/509, 34.8%), followed by *Candida guilliermondii* (136/509, 26.7%), *Candida albicans* (94/509, 18.5%), *Candida glabrata* (41/509, 8.1%), *Candida tropicalis* (39/509, 7.7%), and *Cryptococcus neoformans* (14/509, 2.8%), with low rates of incidence obtained for the remaining infections, which included those caused by *Candida krusei* (5/509), *Candida lusitaniae* (2/509) and *Candida streptococcus* (1/509). **(B)** From 2013 to 2018, changes in the number of IFI patients. **(C)** Compared to other regions, we have a different proportion of fungal species. **(D)** Compared to other regions, we have a lower IFI incidence.

A total of 509 hospitalized patients with IFI were included in this study, with an average ± SD age of 60.1 ± 16.4 (range: 1–93 years old), among which almost 40% were ≥65 years old (202/509, 39.7%), and most were male (322/509, 63.3%). In this study, unlike in other studies, *Candida albicans* was not the most frequently observed cause of infection in these patients (Timsit et al., 2016; Ostrosky-Zeichner and Al-Obaidi, 2017). The most frequent infection agent in our sample was *Candida parapsilosis* (177/509, 34.8%), followed by *Candida guilliermondii* (136/509, 26.7%), *Candida albicans* (94/509, 18.5%), *Candida glabrata* (41/509, 8.1%), *Candida tropicalis* (39/509, 7.7%), and *Cryptococcus neoformans* (14/509, 2.8%), with low rates of incidence obtained for the remaining infections, which included those caused by *Candida krusei* (5/509), *Candida lusitaniae* (2/509) and *Candida streptococcus* (1/509) (Figure 1A). All patients with IFI had at least one associated condition, with the most common concomitant conditions being recent surgery (within 2 weeks) (365/509, 71.7%), followed by hypoproteinaemia (354/509, 69.5%), malignancy (197/509, 38.7%), diabetes (71/509, 13.9%), septic shock (69/509, 13.6%), renal failure (57/509, 11.2%), and AIDS (8/509, 1.6%). In this study, the most common risk factors associated with IFI were prolonged hospitalization (489/509, 96.1%), total parenteral nutrition (404/509, 79.4%), and the presence of catheters (401/509, 78.8%). Supplementary Table 1 contains a summary of the clinical characteristics and inducing factors of candidiasis in the patients included in this study. From 2013 to 2018, the number of IFIs per year was relatively stable (Figure 1B). Compared with those in other regions within a similar time frame, the fungal species observed in our patients showed a different proportion (Figure 1C), and our infection rates were very low (Figure 1D).

### *In vitro* Antifungal Susceptibility Test

The sensitivity test results for the 486 isolates to the five antifungal drugs are shown in Supplementary Table 2. Of the isolates tested, 41/486 (8.4%) were resistant to at least one antifungal agent. For fluconazole, 428/485 (88.2%) of the Candida strains were found to be susceptible. Isolates of C*andida glabrata* showed the highest susceptibility-dose dependence (SDD) to fluconazole (33/41, 80.5%), while the highest resistance was observed with *Candida krusei* (4/5, 80%). It is important to note that 6 isolates (3 *Candida tropicalis*, 2 *Candida glabrata*, and 1 *Candida albicans*) showed cross resistance between fluconazole and voriconazole, 14 isolates (6 *Candida glabrata*, 4 *Candida guilliermondii*, 3 *Candida krusei*, and 1 *Candida albicans*) between fluconazole and itraconazole, 17 isolates (9 *Candida glabrata*, 7 *Candida tropicalis*, and 1 *Cryptococcus neoformans*) showed cross resistance between fluconazole, voriconazole, and itraconazole. And 1 (*Candida krusei*) showed cross resistance between fluconazole, itraconazole, and flucytosine. However, no cross resistance was detected between azoles and amphotericin B.

### Risk Factors for *Candida albicans* and Non-*Candida albicans* Infection

The demographic and clinical characteristics of *Candida albicans*- vs. non-*Candida albicans*-infected patients are listed in Supplementary Table 3. Non-*Candida albicans* infection was more often associated with tumors, central venous catheters, drainage tubes and total parenteral nutrition than *Candida albicans* infection. A larger proportion of *Candida albicans* infection patients were admitted to the ICU, the duration of their ICU stays was longer, they exhibited a greater incidence of septic shock, and lower levels of neutrophils, lymphocytes, and leukocyte were present in these patients.

### Analysis of Risk Factors in Patients With Persistent and Non-persistent Fungal Infections

The demographic and clinical characteristics of patients with persistent vs. non-persistent fungal infections are listed in Supplementary Table 4. Patients with persistent infections were more likely to have renal failure, invasive mechanical ventilation, and multiple/prolonged ICU admissions. Patients with non-persistent fungal infections more often had recent surgery (within 2 weeks) and lower levels of neutrophils, lymphocytes, leukocytes, and PCT.

### Analysis of Risk Factors in Patients With Isolated Fungal Infection and Multiple Fungal Infections

The demographic and clinical characteristics of patients with isolated fungal infections vs. multiple fungal infections are listed in Supplementary Table 5. Patients with isolated fungal infections were more likely to be male, possess tumors, have a recent surgery (within 2 weeks) and have lower levels of lymphocytes, PCT, and CRP. Patients with multiple fungal infections were older; experienced longer lengths of stays and multiple hospitalizations; were more likely to be admitted and stayed longer in the ICU; were more likely to have diabetes, renal failure, septic shock, and persistent fungal infections; and more often used gastric tubes, central venous catheters, and invasive mechanical ventilation.

### Risk Factor Analysis of Patients With and Without Bacteraemia

**Figure 2:**
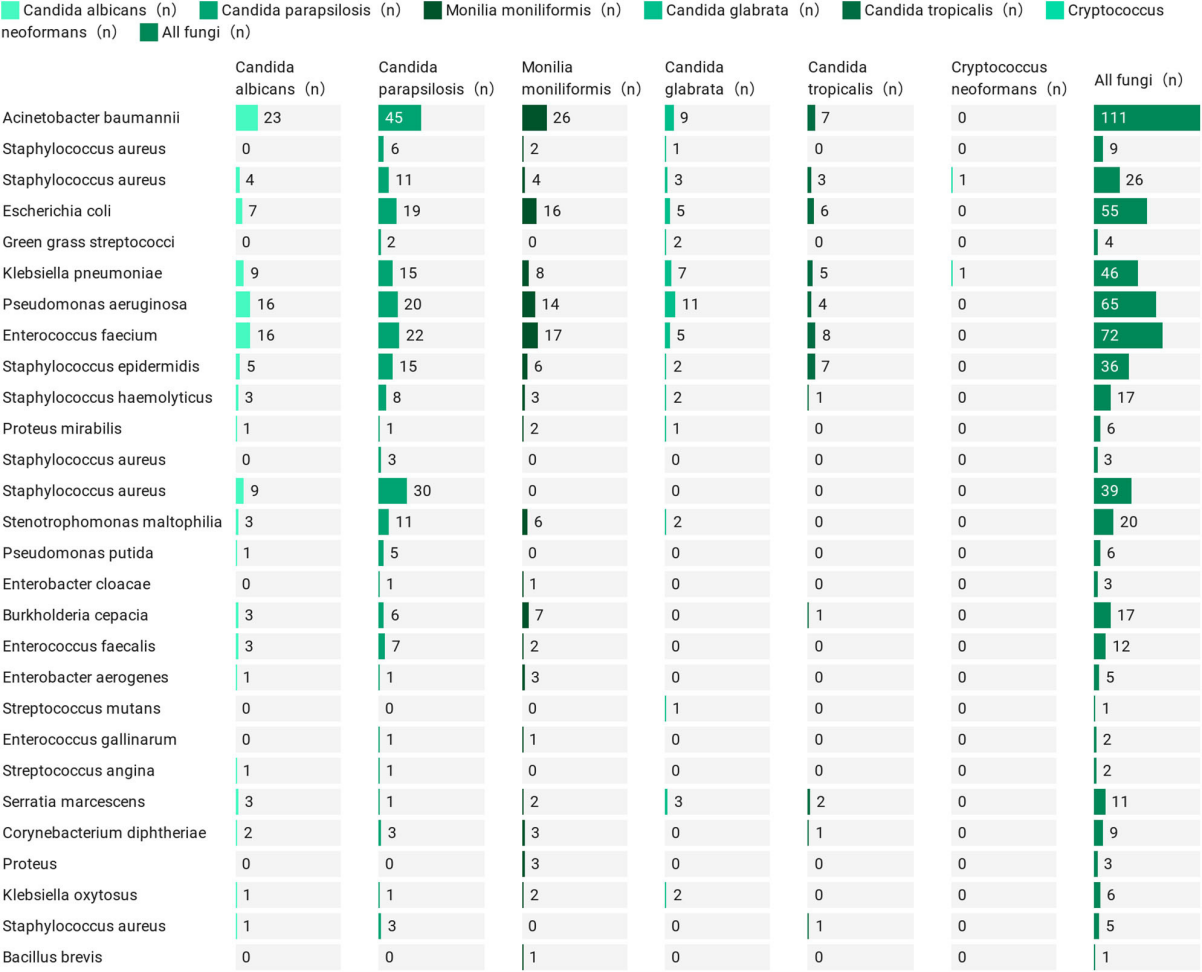
The bacterial and fungal species distribution of bacteremia complicated with IFI.

The demographic and clinical characteristics of patients with vs. without bacteraemia are listed in Table 1. Patients with bacteraemia were older; more often had diabetes, renal failure and septic shock; used invasive mechanical ventilation, catheters, stomach tubes, and central venous catheters; and had persistent fungal infections, as well as multiple/prolonged ICU stays and more frequent hospital admissions. Patients without bacteraemia were more likely to have tumors, recent surgery (within 2 weeks) and lower levels of neutrophils, lymphocytes, leukocytes, total bilirubin, PCT, and CRP. These patients with IFI comprised more than half of the patients with bacterial disease (246/453, 54.3%), with *Acinetobacter baumannii* (111/246, 45.1%) being the most common bacterial infection, followed by *Enterococcus faecium* (72/246, 29.3%), *Pseudomonas aeruginosa* (65/246, 26.4%), *Escherichia coli* (55/246, 22.4%), and *Klebsiella pneumoniae* (46/246, 18.7%) (Figure 2). We cultured 28 species of 591 bacteria from 246 patients, of which 15 were Gram positive (15/28, 53.6%) and 13 were Gram negative (13/28, 46.4%). The specific data are presented in Supplementary Table 6. There were 73 cases of single bacterial infection (73/246, 29.7%) and 173 cases of multiple bacterial infection (173/246, 70.3%). The specific data are presented in Supplementary Table 7. This information provides the basis for the medication employed in the early stage.

**Table 1:** Comparison of bacteremia patients vs. non-bacteremia patients.

**Characteristic**	**IFI patients without bacteremia (*n =* 207)**	**IFI patients with bacteremia (*n =* 246)**	**Statistic**	***P-*value**
Gender (male)	125 (60.37%)	159 (64.63%)	0.867	0.352
Age (years)^a^	59.00 (50.75, 67.25)	63.00 (53.00, 74.25)	−3.363	0.001
Length of stay (days)^a^	25.00 (20.00, 33.00)	45.50 (30.75, 75.50)	−9.578	<0.001
Length of stay in ICU^a^	0.00 (0.00, 2.00)	10.00 (0.00, 30.00)	−10.550	<0.001
Albumin (g/l)^a^	27.4 (23.6, 31.1)	27.3 (23.3, 31.0)	−0.442	0.659
Neutrophils (10^9^/L)^a^	4.85 (3.28, 7.73)	6.53 (4.38, 9.43)	−4.503	<0.001
Lymphocyte (10^9^/L)^a^	0.61 (0.41, 0.89)	0.83 (0.55, 1.20)	−4.813	<0.001
Creatinine (g/l)^a^	59 (47, 75)	59 (41, 90)	−0.023	0.981
Hemameba (10^9/^L)^a^	5.98 (4.30, 9.44)	8.38 (5.77, 11.48)	−5.063	<0.001
Total bilirubin (umol/l)^a^	12.20 (7.10, 21.10)	15.10 (9.50, 28.73)	−3.097	0.002
CRP (mg/l)^a^	39.80 (0.00, 96.10)	93.20 (28.30, 143.75)	−5.726	<0.001
PCT (ng/ml)^a^	0.24 (0.00, 0.71)	0.44 (0.16, 1.79)	−4.516	<0.001
Tumor	119 (57.49%)	96 (39.02%)	15.368	<0.001
Diabetes	21 (10.14%)	47 (19.11%)	7.095	0.008
Pancreatitis	20 (9.66%)	25 (10.16%)	0.032	0.859
Total parenteral nutrition	166 (80.19%)	196 (79.67%)	0.019	0.891
Renal failure	15 (7.25%)	36 (14.63%)	6.141	0.013
Recent surgery (within 2 weeks)	136 (65.70%)	112 (45.53%)	18.463	<0.001
Use immunosuppressants within the past 30 days	18 (8.70%)	15 (6.10%)	1.123	0.689
ICU	58 (28.02%)	168 (28.29%)	72.930	<0.001
Hypoproteinemia	145 (70.05%)	169 (69.98%)	0.060	0.806
Invasive mechanical ventilation	42 (20.29%)	153 (62.20%)	80.517	<0.001
Catheter	150 (72.46%)	210 (85.37%)	11.469	0.001
Stomach tube	101 (48.79%)	166 (67.48%)	16.221	<0.001
Central venous catheter	104 (50.24%)	185 (75.20%)	30.326	<0.001
Drainage tube	133 (64.25%)	168 (68.29%)	0.824	0.364
Septic shock	6 (2.90%)	58 (23.58%)	39.620	<0.001
Multiple hospitalizations within 2 years (>2 times)	113 (54.59%)	167 (67.89%)	8.420	0.004
mortality	19 (9.18%)	85 (34.55%)	40.919	<0.001

a *is described by median and quartile, and the statistic was the Z-value; other items were described as numbers (n–%) and the statistic was the χ^2^-value*.

### Analysis of Risk Factors for Survival and Death in Patients

The demographic and clinical characteristics of survival vs. mortality in patients are listed in Table 2. Patients who survived were often those with tumors, total parenteral nutrition, drainage tubes, recent surgery (within 2 weeks) and lower levels of neutrophils, creatinine, leukocytes, total bilirubin, PCT, and CRP. The patients who died were older, often with renal failure, invasive mechanical ventilation, stomach tubes, central venous catheters, septic shock, persistent fungal infection, multiple/longer lengths of stays in the ICU, and more frequently used immunosuppressants.

**Table 2:** Comparisons of patients that survived vs. died.

**Characteristic**	**Survival patients (*n =* 398)**	**Death patients (*n =* 111)**	**Statistic**	***P-*value**
Gender (male)	244 (61.31%)	78 (70.27%)	3.001	0.083
Age (years)^a^	61.00 (51.50, 70.00)	64.00 (54.00, 78.00)	−2.751	0.006
Length of stay (days)^a^	30.00 (23.00, 49.00)	42.00 (23.00, 75.00)	−2.689	0.007
Length of stay in ICU^a^	0.00 (0.00, 7.00)	14.00 (1.00, 35.00)	−8.233	<0.001
Albumin(g/l)^a^	27.3 (23.5, 30.9)	27.5 (22.9, 32.3)	−0.836	0.403
Neutrophils(10^9^/L)^a^	5.55 (3.79, 8.25)	7.31 (4.35, 10.99)	−3.065	0.002
Lymphocyte(10^9^/L)^a^	0.73 (0.48, 1.08)	0.83 (0.55, 1.18)	−1.529	0.126
Creatinine(g/l)^a^	56 (42, 74)	73 (56, 151)	−3.658	<0.001
Hemameba(10^9/^L)^a^	7.15 (5.00, 10.17)	9.52 (6.12, 12,65)	−3.174	0.002
Total bilirubin(umol/l)^a^	12.95 (7.50, 21.83)	18.60 (9.80, 35.70)	−4.906	<0.001
CRP(mg/l)^a^	56.75 (0.00, 104.25)	93.50 (30.80, 147.00)	−4.906	<0.001
PCT(ng/ml)^a^	0.23 (0.00, 0.75)	0.73 (0.24, 2.53)	−5.701	<0.001
Tumor	211 (53.01%)	33 (29.73%)	18.856	<0.001
Diabetes	51 (12.81%)	20 (18.02%)	1.958	0.162
Pancreatitis	38 (9.55%)	11 (9.91%)	0.013	0.909
Total parenteral nutrition	329 (82.66%)	75 (67.57%)	12.080	0.001
Renal failure	32 (8.04%)	25 (22.52%)	18.306	<0.001
Recent surgery (within 2 weeks)	241 (60.55%)	39 (35.14%)	22.657	<0.001
Use immunosuppressants within the past 30 days	21 (5.28%)	13 (11.71%)	5.766	0.016
ICU	156 (39.20%)	82 (73.87%)	41.926	<0.001
Hypoproteinemia	283 (71.11%)	71 (64.55%)	1.756	0.185
Invasive mechanical ventilation	132 (33.17%)	75 (65.77%)	38.347	<0.001
Catheter	313 (78.64%)	88 (79.28%)	0.021	0.885
Stomach tube	213 (53.52%)	77 (69.37%)	8.897	0.003
Central venous catheter	233 (58.54%)	79 (71.17%)	5.834	0.016
Drainage tube	277 (69.60%)	58 (52.25%)	11.607	0.001
Septic shock	26 (6.53%)	42 (37.84%)	73.487	<0.001
Multiple hospitalizations within 2 years (>2 times)	232 (58.29%)	78 (70.27%)	5.230	0.022

a *is described by median and quartile, and the statistic was the Z-value; other items were described as numbers (n–%) and the statistic was the χ^2^-value*.

### Prognostic Factors Associated With Death

In patients with invasive fungal infections, with death as the dependent variable (0 = survival, 1 = death) in a univariate analysis, statistically significant effects were obtained with regard to age; length of hospital/ICU stay; levels of neutrophils, creatinine, leukocytes, total bilirubin, PCT, and CRP; presence of tumors; total parenteral nutrition; renal failure; recent surgery; use of immunosuppressants within the past 30 days; ICU admission; invasive mechanical ventilation; stomach tubes; central venous catheters; drainage tubes; septic shock; multiple hospitalizations; and persistent fungal infections as independent variables with multivariate logistic regression analysis performed by the conditional method. These results revealed that length of ICU stay, renal failure, levels of neutrophils, and total bilirubin, use of immunosuppressants within the past 30 days, ICU admission, stomach tube placement, recent surgery, drainage tube placement, and septic shock were risk factors for death in IFI patients (Table 3).

**Table 3:** Multivariate Logistic regression analysis of prognostic factors associated with death.

	**β**	$S_{\bar{x}}$	**Waldχ^**2**^**	***P-*value**	***OR***	**95%*CI***
Length of stay in ICU	0.012	0.005	5.699	0.017	1.012	1.002~1.002
Neutrophils	0.074	0.027	7.745	0.005	1.077	1.022–1.134
Total bilirubin	0.007	0.003	4.379	0.036	1.007	1.000–1.013
Renal failure	0.978	0.345	8.050	0.005	2.658	1.353~5.223
Recent surgery (within 2 weeks)	−1.022	0.318	10.355	0.001	0.360	0.193~0.671
Use immunosuppressants within the past 30 days	1.267	0.437	8.404	0.004	3.549	1.507~8.359
ICU^a^	0.802	0.347	5.338	0.021	2.230	1.129~4.405
Stomach tube	0.753	0.352	4.568	0.033	2.123	1.064~4.233
Drainage tube	−0.970	0.329	8.720	0.003	0.379	0.199~0.722
Septic shock	1.994	0.334	35.648	<0.001	7.344	3.817~14.131

a *there was a negative correlation between the value and significance*.

### Comparison of Serum Marker Levels Between Patients Who Survived and Those Who Died

**Figure 3:**
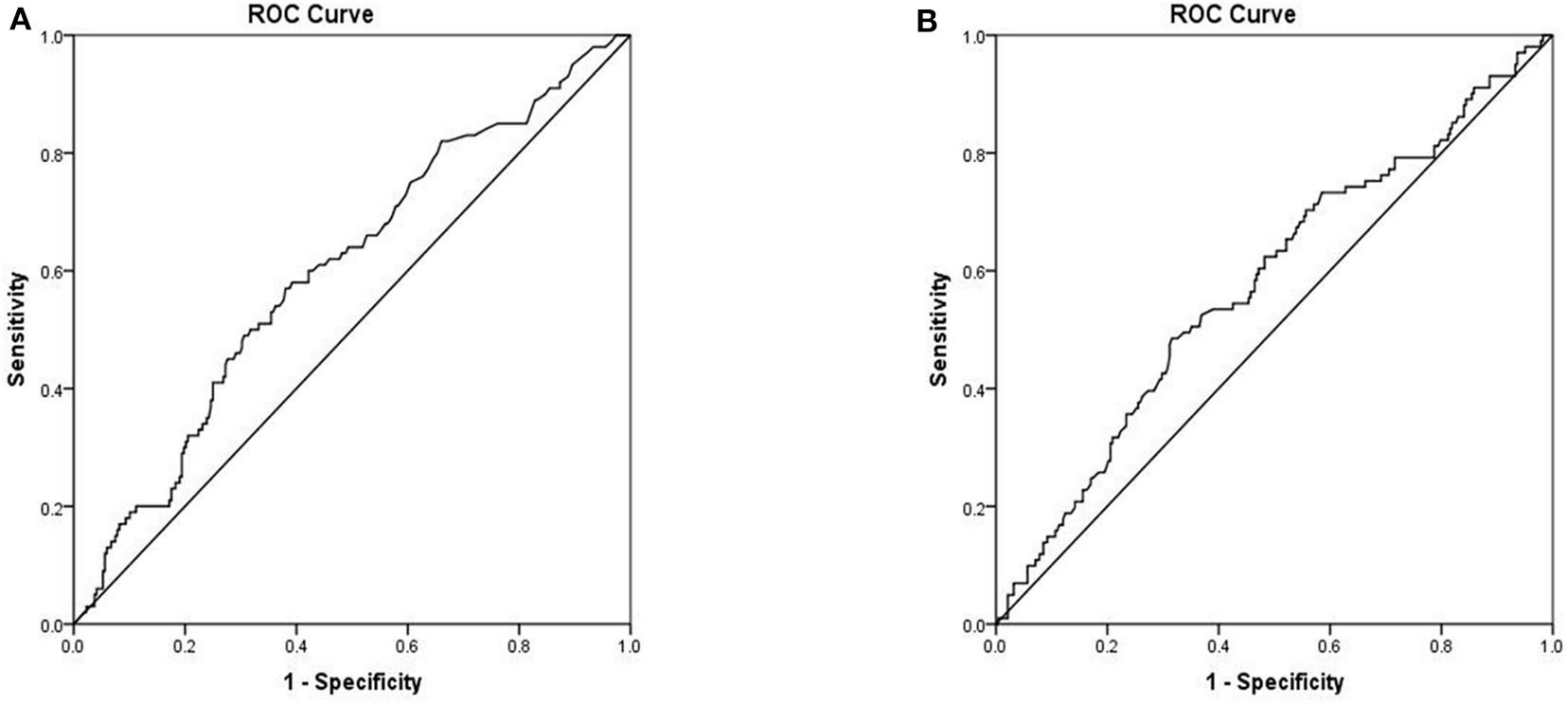
Receiver operation characteristics (ROC) analysis of two independent mortality risk factors **(A)** PCT **(B)** CRP.

To assess the ability of serum markers to serve as prognostic indicators for IFI, ROC analysis was performed using significantly different immunological parameters between patients who survived and those who did not. Serum PCT (AUC 0.601, 95% CI 0.536–0.665, *P* = 0.003) possessed a higher prognostic value in IFI patients than other parameters (Figure 3A). The critical serum value for survival vs. death was 0.72 ng/ml, with a sensitivity of 57.0%, and a specificity of 61.9%. Serum CRP (AUC 0.578, 95% CI 0.512–0.644, *P* = 0.020) was also found to be valuable in predicting the prognosis of IFI (Figure 3B). We also tested BDG within ROC analysis and found that BDG (AUC 0.519, 95% CI 0.423–0.616, *P* = 0.693) failed to be a significant factor for IFI prognosis (Table 4).

**Table 4:** Receiver operating characteristics curve analysis of immune parameters predicting death.

	**AUC**	**95%CI**	***P-*value**	**Optimal threshold value**	**Sensitivity**	**Specificity**
BDG	0.519	(0.423–0.616)	0.693	10.88	78.0%	27.0%
PCT	0.601	(0.536–0.665)	0.003	0.72	57.0%	61.9%
CRP	0.578	(0.512–0.644)	0.020	111.50	48.5%	68.4%
CD4	0.517	(0.399–0.635)	0.780	154.50	76.9%	33.9%
CD8^a^	0.509	(0.389–0.629)	0.880	143.00	48.7%	64.3%
CD3^a^	0.500	(0.376–0.624)	0.997	309.00	43.6%	71.4%
CD4/CD8	0.535	(0.501–0.655)	0.568	0.96	41.0%	73.2%

a *there was a negative correlation between the value and significance*.

## Discussion

To better understand the clinical characteristics and prognostic factors of IFI, we retrospectively surveyed 509 IFI patients with different demographic and risk factors as well as clinical and laboratory characteristics. It had been reported previously that *Candida albicans* represents the most common invasive fungal infection species (Fu et al., 2017), but our current data show that the proportion of non-*Candida albicans* infections exceeds that of *Candida albicans* infections, with *Candida parapsilosis* being the most common Candida species. Such differences may be attributable to the epidemiology of IFI, which can vary depending on patient age, geographic area, medical practice and antifungal use. According to our results, the overall incidence of IFI in Northeast China (0.07/1,000) was substantially lower than that reported in Italy (1.5/1,000), Israel (0.61/1,000), and the United States (0.3/1,000) as well as in other regions within China, such as Chongqing (0.32/1,000) and Beijing (0.55/1,000) (Wang et al., 2014; Barchiesi et al., 2016; Eliakim-Raz et al., 2016; Alothman et al., 2017). When assessing known potential risk factors for IFI (Supplementary Table 1), we found that the most common risk factor was prolonged hospitalization (489/509, 96.1%), suggesting that IFI may be associated with nosocomial infections resulting from prolonged hospitalization. This observation is consistent with previous studies on fungal infections (Pu et al., 2017). It seems likely that prolonged hospitalizations may increase exposure to fungal pathogens, which are abundant in hospital settings and difficult to eradicate (Abreu et al., 2013). However, as long-term hospitalization is often not an independent risk factor, it is important for the clinician to consider and test for invasive fungal infections in patients who have been hospitalized for longer than 10 days. Other risk factors associated with IFI include total parenteral nutrition (79.4%), catheters (78.8%), and recent surgery (71.7%) (Supplementary Table 1). The common risk factors observed in this study were similar to those reported by others (Zaoutis et al., 2005; Wang et al., 2014; Barchiesi et al., 2016; Jia et al., 2018).

The main clinical features shown by the 509 IFI patients in this study were an average age of 60.1 years, with almost 40% being > 65 years old (202/509, 39.7%), a greater number of males (322/509, 63.3%), and more who had continuously used antibiotics for > 2 weeks (289/509, 56.8%). Overall, these findings are similar to those of previous studies in China (Cheng et al., 2005; Shigemura et al., 2014; Pu et al., 2017). In addition, we observed that patients with hypoproteinaemia (354/509, 69.5%) were more likely to present IFI. Such patients may have weakened immune function and/or other diseases, which would increase their susceptibility to infection by fungal pathogens (Batzlaff and Limper, 2017).

In our study, most IFI events occurred in the surgical ward (331/509, 65.0%), but both the medical and surgical wards had a significant number of patients who had been admitted to the ICU during their hospitalization, suggesting that the basic state of the patients was associated with IFI infection risk. A number of factors may contribute to these differences, including the size of the department, patient population and size of the hospital. Thus, specific predictors of IFI may differ among hospitals, as well as in different wards within the hospital. In our study, the majority of surgical patients underwent abdominal surgery, which may damage the gastrointestinal barrier. Such a procedure can lead to skin contamination at the site of vascular insertion, making these patients more susceptible to IFI (Moreno Elola-Olaso et al., 2012; Prieto et al., 2014). In addition, IFI patients who had been admitted to the ICU during hospitalization had significantly higher mortality rates than those in the surgical ward. Several factors may influence such differences, including patient age, severity of primary disease and presence of various comorbidities (Timsit et al., 2016; Ostrosky-Zeichner and Al-Obaidi, 2017). Therefore, infection control measures should be individually designed and implemented for specific use within each different ward to reduce the incidence of IFI. We also found that resistance to azoles was observed mainly in *C. tropicalis* and *C. glabrata* isolates, a result that was similar to that previously reported (Wang et al., 2016). These findings have important implications for the selection of empirical antifungal agents in patients suspected of candidiasis.

To our knowledge, the findings presented here are the first to report that more than half of these IFI patients had bacteraemia (246/453, 54.3%), which should alert and introduce a cautionary note to clinicians treating IFI patients. *Acinetobacter baumannii* (111/246, 45.1%) was the most common co-infecting bacterium, followed by *Enterococcus faecalis* (72/246, 29.3%), *Pseudomonas aeruginosa* (65/246, 26.4%), *Escherichia coli* (55/246, 22.4%), and *Klebsiella pneumoniae* (46/246, 18.7%). Of the 246 patients with IFI concomitant with bacteraemia, 73 (29.7%) presented with a single bacterial infection, and the remaining 173 (70.3%) presented with multiple bacterial infections. These rates of bacterial infection provide a basis and guidance for the treatment of bacterial infections in IFI patients. Fungi and bacteria can produce both synergistic or antagonistic effects, and *Acinetobacter baumannii* can use ethanol produced by yeast as a carbon source to enhance its growth (Smith et al., 2004; Garsin and Lorenz, 2013). *Pseudomonas aeruginosa* often synergizes with fungi, with the result that *Aspergillus fumigatus* can enhance elastase production in co-cultures with *Pseudomonas aeruginosa*, leading to a poor prognosis in IFI patients (Smith et al., 2015; Pekmezovic et al., 2016). Fungal 1,3-glucan has been shown to increase the tolerance of *Escherichia coli* to ofloxacin in *Escherichia coli/Candida albicans* biofilms (Tang et al., 2018). The mortality rates of IFI patients with bacteraemia (85/246, 34.6%) were significantly greater than those of patients without bacterial infection (19/207, 9.2%), which is a noteworthy factor with regard to the clinical treatment of IFI patients presenting with this specific condition. Another clinical mortality complication related to fungal infections was septic shock (13.6%). This situation may be related to the individual patient's condition along with a lack of timely antifungal treatment, leading to a poor prognosis in these patients.

The results of our study also suggest that serum CRP and PCT levels may be reliable predictors of mortality. These findings are consistent with some recent studies and indicate the potential of these markers for prognosis (Giacobbe et al., 2017; Tang et al., 2018; Cortegiani et al., 2019). While the commonly used diagnostic biomarker for IFI is an elevation in serum BDG levels (León et al., 2012; Posteraro et al., 2016), in our study, we found that elevated BDG was not a reliable indicator of prognosis. Therefore, we suggest that serum PCT and CRP can more effectively serve as predictors of survival and death in these patients.

This study has some limitations. First, as it was conducted in only one hospital; thus, it cannot be determined whether these results can be extrapolated to IFI cases elsewhere. We hope to expand upon this research at other sites. Second, as the identified fungal species were confirmed using the fungal culture method, the possibility of an error in the identification of these fungi cannot be completely ruled out. Third, the severity of patients was not graded prior to treatment, which may introduce a confounding factor in the analysis of treatment outcomes.

Despite these limitations, this study represents the first comprehensive description of pathogens, clinical characteristics, and potential risk factors for mortality in IFI patients within this region. Such information can provide new insights and protocols for improvements in IFI management.

## Conclusions

In this study, we identified clinical characteristics and risk factors associated with mortality rates in patients with IFI, along with the first review of epidemiological information on IFI patients with bacteraemia. *Candida parapsilosis* was found to comprise the main type of infection, followed by *Candida guilliermondii* and *Candida albicans*. Risk factors, such as longer ICU stays, renal insufficiency, immunosuppressive agents, levels of neutrophils and total bilirubin, ICU admission rates, gastric tube placement, and septic shock, were independently associated with mortality. Serum PCT and CRP levels can serve as effective biomarkers for the prognosis of IFI. These findings will greatly aid clinical health care workers with regard to the identification, prevention and treatment of IFI in hospitalized patients.

## Data Availability Statement

All datasets generated for this study are included in the article/Supplementary Material.

## Ethics Statement

Ethical review and approval was not required for the study on human participants in accordance with the local legislation and institutional requirements. Written informed consent from the patients was not required to participate in this study in accordance with the national legislation and the institutional requirement.

## Author Contributions

YL and XGu designed and supervised the study. YL interpreted the data and prepared the manuscript with support from YG. YL was organized the data acquisition as well as data analysis. All authors gave the approval of the final version manuscript.

## Conflict of Interest

The authors declare that the research was conducted in the absence of any commercial or financial relationships that could be construed as a potential conflict of interest.
